# Efficacy and safety of electroacupuncture at auricular concha region in promoting of rehabilitation of ischemic stroke patients with upper limb motor dysfunction: A study protocol for a randomized pilot trial

**DOI:** 10.1097/MD.0000000000028047

**Published:** 2022-04-15

**Authors:** Yilin Liu, Liping Zhang, Sanrong Wang, Lu Long, Qianwen Zang, Gongwei Jia

**Affiliations:** a Chongqing Medical University, 1 Yixueyuan Road, Yuzhong District, China; b Department of Rehabilitation Medicine, The Second Affiliated Hospital of Chongqing Medical University, 76 Linjiang Road, Yuzhong District, China.

**Keywords:** auricular acupuncture, electroacupuncture, stroke, upper limb, vagus nerve

## Abstract

**Introduction:**

Ischemic stroke (IS) is the one of the most severe neurological disease, survivors may live with upper limb motor dysfunction (ULMD) resulting in heavy social and economic burden. Nowadays, there are few approaches to promote the rehabilitation of ULMD. Auricular acupuncture (electroacupuncture [EA]) has long been used in the treatment of neurological disorders in China. This treatment has become an attractive treatment option due to its low cost, portability, minimal side effects, and ease of use in clinical and operational environments. However, its efficacy and safety in consciousness recovery remain to be proved.

**Methods:**

A total of 80 IS patients with single upper limb motor function impairment will be recruited in the trial and randomized into EA or control groups. Patients in the control group will receive routine conventional treatment alone while patients in the EA group will receive EA treatment for 3 consecutive weeks based on routine conventional treatment. Baseline evaluation was carried out on day of enrollment, post-treatment evaluation was carried out 14 and 21 days after enrollment, and the 2 groups were follow-ups in 3 and 6 months after the end of the trial. The efficacy will be assessed with the changes in the upper limb Fugl–Meyer assessment, Wolf motor function test, action research arm test, active range of motion, and Barthel index. The safety of EA will be estimated by monitoring the incidence of adverse events and changes in vital signs during the study period. Analysis of feasibility will be descriptive and the change in outcome measures between groups will be analyzed using an independent sample *t* test.

**Discussion:**

This study tried to narrow the evidence gap on the efficacy of EA at the auricular on the recovery of ULMD in patients with IS. The results of this trial will provide strong evidence for the efficacy and safety of EA of auricular concha region stimulation for IS patients.

**Trial registration:** This trial has been registered at the Chinese Clinical Trial Registry, numbered ChiCTR2100049678.

## 1 Introduction

Ischemic stroke (IS) is a leading cause of mortality and morbidity worldwide, which associated with the disability and increases the social economic burden.^[1]^ The upper limb motor dysfunction (ULMD) is widespread around the patients and has been thought to be common after IS, which is considered to be in need of treatment.^[2]^ Thus far, approximately 85% of patients with ULMD after IS have arm weakness as well as 60% of survivors with non-function arms cannot recover function within 6 months.^[3,4]^ At present, comprehensive rehabilitation programs had been being used to the rehabilitation of ULMD such as neuromuscular electrical stimulation, forced exercise therapy, occupational therapy, imagination therapy, and rehabilitation robots.^[5]^ However, the implementation of the above-mentioned treatment strategies for ULMD should be moderate.^[6]^ New and more effective treatment methods need to be considered.

Acupuncture has been used for over 2000 years in China. As a complementary and alternative management for neurological disease, including IS, acupuncture is popular all over the world for features of easy operation and safety.^[7]^ Auricular acupuncture (AA) is an important part of Chinese acupuncture. The Yuan Dynasty Weisheng Baojian believed: “five viscera and six bowels, twelve meridians and collaterals coverage in the ear.”^[8]^ AA therapy can not only adjust the mind directly, but also cooperate with the ear or indirectly stimulate the 5 viscera (heart, liver, lung, spleen, and kidney) by stimulating the auricular concha area (ACR), so as to realize the harmoniousness of heart-brain and balance Qi and blood.^[9]^ Animal studies found that electroacupuncture (EA) at ACR (EA-ACR) had significant effects in the management of primary hypertension,^[10]^ diabetes,^[11]^ and partial epilepsy.^[12]^ EA-ACR is a noninvasive procedure that requires a portable EA device with less side effects. However, clinical evidence on its efficacy is controversial due to problems concerning the study design, including small sample size, high drop-out rate, lack of proper control, and high/unclear risk of bias.

Therefore, we intend to evaluate the efficacy and safety of EA-ACR in the recovery of ULMD in patients with IS. Furthermore, we hope that this trial will provide high-quality evidence for the application of EA-ACR in the management of IS patients as well as to establish a standard acupuncture procedure for clinical practice.

## 2 Methods

### 2.1 Study design

Here, we design a blinded, randomized controlled trial, 80 patients with ULMD after IS were recruited by clinicians. The EA treatment protocol was determined according to reference and results of previous research on EA treatment for ULMD. All participants will go through a standardized interview and be provided with details of the study. The acupuncturists who deliver treatments for EA groups are registered with the Ministry of Health of the People's Republic of China as Chinese medicine practitioners and have >10 years of clinical experience.

**Figure 1:**
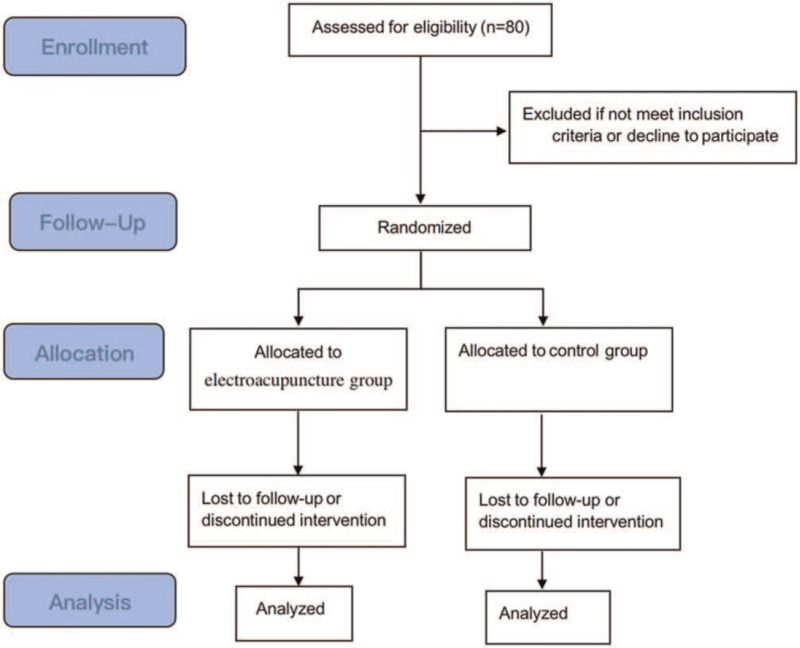
Flow chart of the study procedure.

In addition to routine treatment, patients in the EA group will receive EA treatment for 3 consecutive weeks. Patients in the control group will receive routine treatment alone. The research process is shown in Fig. 1.

### 2.2 Sample size

The sample size was calculated according to the following formula:


$$\text{N}=\frac{{\left[{\text{u}}_{\alpha }\sqrt{2\text{p}(1-\text{p})}+{\text{u}}_{2\beta }\sqrt{{\text{p}}_{1}(1-{\text{p}}_{1})+{\text{p}}_{2}(1-{\text{p}}_{2})}\right]}^{2}}{{\left({\text{p}}_{1}-{\text{p}}_{2}\right)}^{2}}$$

when the significance level is *α* = 0.05, *β* = 0.10, N is the required sample size for each group. Calculated by the formula: N = 32. There may be the probability of missing to follow-up and termination of various reasons among the subjects. Thus, allowing for 10% to 15% of attrition, the sample size should be adjusted N = 40, that is, each group 40 patients, so the sample size of the 2 groups of patients is determined to be 80 cases.

### 2.3 Inclusion criteria

(1) Aged 18 to 80 years old;
(2) have cerebral infarction with a first onset and onset >6 months;
(3) the previous physical activity was normal, and there were different degrees of hemiplegia in admission;
(4) vital signs are stable;
(5) Mini-Mental State Examination (MMSE) score ≥22 and compliance with the interventions;
(6) can take the secondary prevention drugs for cerebral infarction and patients with routine rehabilitation training;
(7) written informed consent of the patient or their legal guardian has been obtained.

### 2.4 Exclusion criteria

(1) Hemorrhagic cerebral infarction, lacunar infarction;
(2) relatively severe systemic diseases (severe cardiovascular disease, respiratory, digestive, urinary system dysfunction, and hematopathy);
(3) malignant tumors or various infectious diseases;
(4) patients with locomotor system and other diseases affect muscle strength assessment;
(5) patients who cannot be combined with rehabilitation assessment.

### 2.5 Participant recruitment

Patients who meet our eligibility criteria will be screened consecutively from Department of Rehabilitation Medicine, The Second Affiliated Hospital of Chongqing Medical University. Researchers will screen the list of patients every other day, continue to recruit and monitor recruitment. Eligible patients will be screened, oral and written information about the study will be obtained from the researchers later. After signing the informed consent form, demographic and clinical characteristics will be measured (baseline assessment, t0). Weekly recruitment statistics will be discussed to improve the recruitment process. On this basis, if necessary, the research team discussed updates to recruitment and retention strategies.

### 2.6 Randomization and allocation concealment

The randomization sequence will be generated by computer using SPSS 26.0 (IBM, USA). All participants who meet the inclusion criteria will be randomly assigned to an intervention group or control group (40 cases each) at a 1:1 ratio. Opaque computer-generated sealed envelopes will be produced to cover the distribution. The envelope will be serially numbered with a serial number on the outside and will contain distribution information on the inside. Upon completion of baseline assessment and written informed consent, the subject will be enrolled in the study, at which time the envelope will be opened. Participants will be assigned to one of the two groups and receive related interventions. The random distribution sequence and the opaque sealed envelope will be kept separately by 2 specific researchers.

### 2.7 Blinding

Each participant was initially interviewed and evaluated by a clinician, and the evaluation was then confirmed by another well-trained and experienced clinician. The evaluators, outcome assessors, and statisticians will be blinded to the group allocation and all of them will work independently and separately. It is a pity that patients could not be blinded in this study.

### 2.8 Interventions

#### 2.8.1 Selected principles of acupuncture point

We selected the auricular acupoints of The Five Viscera (Yin Organs) and Six Bowels (Yang Organs), tissues and organs related to the etiology and mechanism of stroke in traditional Chinese medicine (TCM), as well as the relevant auricular acupoints of the pathogenesis sites and associated systems in modern medicine, including CO15 (Xin), AT4 (Pizhixia), CO10 (Shen), and CO12 (Gan). Acupoint positioning is based on National Standards of People's Republic of China: name and location of ear acupoints (GB/T 13734-1992).^[13]^

#### 2.8.2 Treatment group

**Figure 2:**
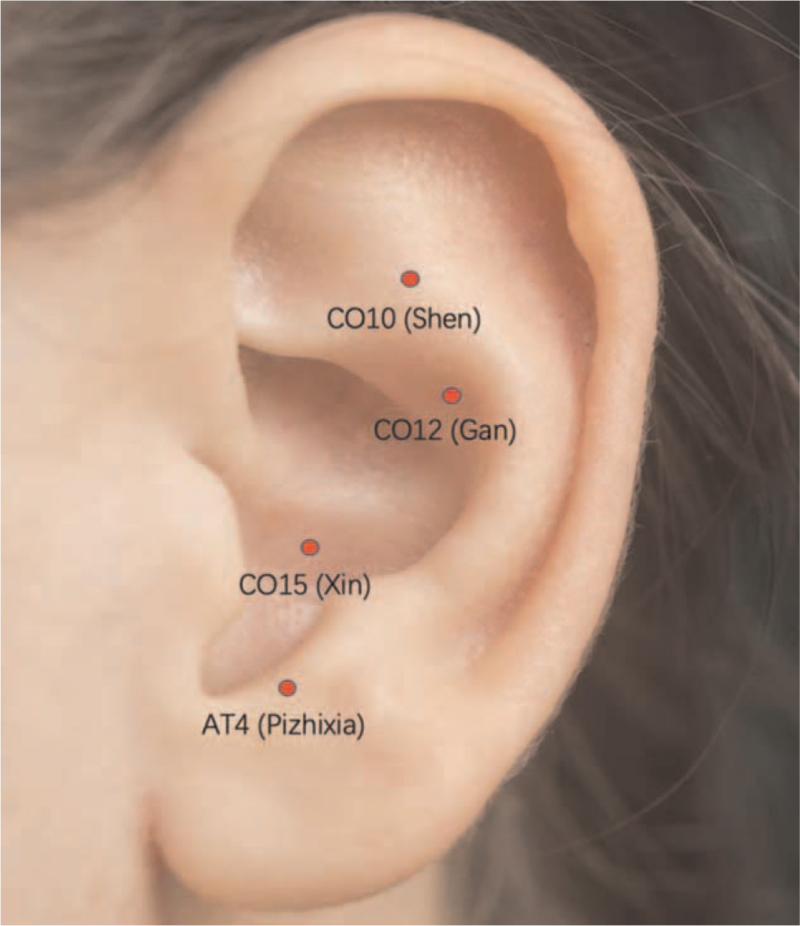
Schematic sites of acupuncture points in EA group.

Figure 2 shows the schematic sites of acupuncture points in EA group. Sterilized needles (the acupuncture needles of 0.30 mm in diameter and 25 mm in length [Suzhou Hualun Medical Appliance Co. Ltd., China]) will be used. Acupuncture is inserted vertically, inserted at a depth of about 0.1 to 0.3 cm, and the electrical stimulation device is connected after Deqi sensation. The parameters were selected as follows: 20 Hz square wave, current intensity 0.5 mA, each lasting 30 seconds, stimulating once every 5 minutes. The participant could withdraw from the trial if he could not tolerate the stimulation. Stimulation was performed for 30 minutes per day for 3 consecutive weeks.

#### 2.8.3 Control group

Participants who are assigned to the control group will receive routine rehabilitation training, which is customized according to the patient's abilities and applied to the limbs and trunks, involving postural control, turn over regularly, joint mobility training, muscle strength training, and always at the upper limit of their capacity.

#### 2.8.4 Follow-up

All patients will receive evaluation at 0 days after enrollment, 14 and 21 days after post-treatment, and 3 and 6 months at the end of the trial. Scoring data are collected, as described in Table 1.

**Table 1:**
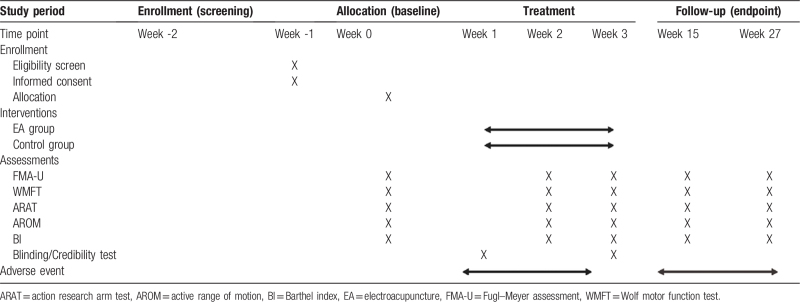
Schedule of enrollment, interventions, and assessments.

### 2.9 Outcomes

#### 2.9.1 Primary outcome

##### 2.9.1.1 Upper limb Fugl–Meyer assessment (FMA-U)

FMA-U was used to evaluate 33 items of motor function and reflex activity of affected lower limb joints. It remains 9 items including reflex, shoulder, elbow, wrist, hand, etc by a 3-point system (maximum motor score, 66 points). To observe the long-term effects, FMA-U was evaluated 6 months after the intervention.

#### 2.9.2 Secondary outcomes

##### 2.9.2.1 Wolf motor function test

Wolf motor function test (WMFT) focuses on evaluate the rehabilitation scale of ULMF after stroke. The design of the WMFT ranges from simple to complex, including proximal and distal joints, to test the quality and speed of the upper limb movement. It can quantitatively evaluate the exercise ability of the patient's upper limbs by timing single joint movement, multi-joint movement, and functional activity and evaluating the quality of exercise. WMFT consists of 15 projects, and 6-point scale ranging from 0 to 5 (normal).

##### 2.9.2.2 Action research arm test

Action research arm test has a total of 19 test items, divided into 4 groups of subscales: Grasp, Grip, Pinch, and Gross Movement. Action research arm test has been regarded as an effective method for assessing specific arm activity limitations which can assess the participant's ability to handle objects of different sizes, weights, and shapes.

##### 2.9.2.3 Active range of motion

Measuring the active range of motion of a joint is to investigate the influence of the participant's muscle contraction force on the joint activity change values.

##### 2.9.2.4 Barthel index

Barthel index is a 3-level evaluation that measures independence in basic activities of daily living following stroke-induced hemiplegia. A normal score is 100. Ability of daily life disorders can be divided into mild (61–99 points), moderate (41–60 points), severe (1–40 points), and completely dependent (0 points).

### 2.10 Adverse events

Patients’ legal representatives will be informed of potential adverse events from acupuncture prior to signing the consent form. Any adverse events, including acupoint hematoma, infection, and apostasies, will be recorded by the researchers. In case of severe adverse events occurring, acupuncture intervention will be ceased immediately and proper treatment will be provided. All severe adverse events will be reported to the principle investigator and the ethics committee within 48 hours. Patients will be followed up for 6 month after the trial.

### 2.11 Data management and monitoring

Efficacy evaluation and data collection will be carried out by members of the research team other than the operators. The upper limb function of stroke patients was evaluated at the time of enrollment and after each treatment. Researchers managing data will receive training before registration. The evaluator will be responsible for the acquisition of participant information and result evaluation during the study period. First, the evaluator uses the paper version of the evaluation form to record the data, and then enters the data into the excel form for management. The original case report form and all other forms (including the consent form) will be safely archived in the Department of Rehabilitation Medicine, The Second Affiliated Hospital of Chongqing Medical University. Only researchers from our research team can access the final test data.

The composition of data monitoring committee is independent of researchers and has no competing interests; it will check original case report forms, monitor the quality and completeness of data, verify records of adverse events, and ensure that the research complies with the principles of the protocol. Periodic reviews are conducted every 2 months.

### 2.12 Statistical analysis

SPSS 26.0 (IBM SPSS, Chicago, IL) will be used to analyze the data. Quantitative data will be presented as mean ± SD. For comparisons of baseline characteristics, the Chi-square test was used for categorical variables, and the Kruskal–Wallis test was used for continuous variables. The characteristics of participants with missing data will be compared between the 2 treatment groups (healthy vs unhealthy outcomes). Inconsistencies will be discussed until a consensus is reached, and further analysis will identify research issues/problems. An independent sample *t* test was used for comparisons of the change in outcome measures between groups. If the data were not normally distributed non-parametric, Wilcoxon and Mann–Whitney *U* tests were applied. The characteristics of subjects without complete follow-up and factors associated with dropout will be analyzed. *P* values of <.05 will be considered statistically significant.

### 2.13 Withdrawal and dropout

According to the Declaration of Helsinki, all participants will be respected and can withdraw from the study at any time for any reason. Personal information about participants will be collected and kept confidential. Participants will be excluded from the study if they use a treatment combination prohibited by the study protocol, withdraw their consent, or stop communicating. The investigator will report on the withdrawal and the reasons for the withdrawal and obtain measures of the time of the last treatment and possible outcomes.

### 2.14 Ethics and dissemination

This study has been approved by the Institutional Review Board and Hospital Research Ethics Committee of Chongqing Medical University (approval NO. 2020216) and was conducted at the Department of Rehabilitation Medicine, the Second Affiliated Hospital between March 2021 and June 2022. Our protocol named “Efficacy and safety of electroacupuncture at auricular concha region in promoting of rehabilitation of ischemic stroke patients with upper limb motor dysfunction: a study protocol for a randomized pilot trial” was registered on http://www.chictr.org.cn/showprojen.aspx?proj=131569 with registration number ChiCTR2100049678. If there are any protocol modifications, we will report to the Ethics and Research Committee for approval. All patients will be recruited from the Department of Rehabilitation Medicine with signed informed consent from their legal representatives prior to their enrollment. Participant information will be protected. All experimental data will be stored in a secure storage area with access limited to the researchers alone. The results of the study will be published as peer-reviewed manuscripts and congress presentations, communicated with patients, and the clinical community.

### 2.15 Quality control

We also established an independent quality control team to be responsible for patient inclusion, informed consent, and randomization. Training includes patient recruitment, disease assessment, data collection, and follow-up to minimize selection bias. In the process of data collection, the statisticians in this study were not clear about the grouping situation and its significance, so as to reduce unnecessary communication with the subjects and eliminate the influence of the above interference factors in the statistical analysis. The quality control group is also responsible for long-term patient communication and health education. The above measures can improve patient compliance and reduce the failure rate of follow-up.

## 3 Discussion

IS, a severe disease which not only brings along physical pains in patients, but also results in heavy social and economic burden, accounts for 80% of 12.42 million stroke patients aged 40 or older in China in 2016.^[14]^ Without promptly and adequately diagnosed and treated, IS patients will suffer serious damage, which can lead to a decrease in quality of life and even disability in severe cases.^[15]^ The common and widely recognized impairment caused by IS is ULMD, which can be regarded as a loss or limitation of function in upper limb muscle control or movement or a limitation in mobility.^[16]^

AA is a form of acupuncture that originated in China.^[17]^ The practice of AA is mentioned in the Yellow Emporer's Classic of Internal Medicine, one of China's oldest medical classics. In this book, Yang meridian, 5 viscera and 6 bowels and kidney are documented as directly connected to the ear.^[18]^ The heart is the organ similar to the monarch and is responsible for spirit and mental activity.^[19]^ The heart, located where the “spirit” (shen) resides, opens into the ear and governs the blood according to TCM.^[20]^ “Regulating the heart, treating collaterals from the heart, nourishing the heart and dredging collaterals."^[21]^ CO15 (Xin) was often used as corresponding channel point selection for sequelae of stroke.^[22,23]^ EA at CO15 (Xin) could activate the cervical vagus nerve, increasing the discharge activity of the nucleus tractus solitarius of the ascending nucleus of the vagus nerve.^[24]^ Horseradish peroxidase (HRP) nerve tracer was injected into the auricle area for tagging neurons, which could be found that tagged neurons appear in nuclei such as the nucleus tractus solitarius and the dorsal motor nucleus of the vagus nerve.^[25]^ Study showed that non-invasive electrical stimulation of the ACH, aiming to activate vagal afferences, appeared to be beneficial to the recovery of upper limb motor function in subacute IS patients.^[26]^ AT4 (Pizhixia) can regulate the central nervous system, inhibit the excitability of the cerebral cortex and autonomic nerve center, which have the functions of relieve mental strain and regulating viscera.^[22]^ AT4 (Pizhixia) is commonly used to treat stroke also have a certain degree of efficacy in relieving post-stroke pain and reducing inflammatory response.^[27]^ The Kidney was the “Sea of Marrow” and relate to the brain in TCM. The Kidney energetic organ system is in charge of storing Essence or Jing; abundant Essence results in normal hearing as the Ling Shu explains, “… depletion of Essence gives rise to deafness.”^[28]^ The liver controls the smooth flow of Qi throughout the body. If wood imbalances are not properly addressed, Qi stagnation can develop. Since blood circulation depends on Qi circulation, blood stasis can result. Sudden onset of ear pain or tinnitus, sudden deafness can result from blood stagnation due to Qi stagnation.^[29]^ Herein, our study selected EA to stimulate CO15 (Xin), AT4 (Pizhixia), CO10 (Shen), and CO12 (Gan) based on preliminary foundation and clinical research results.

The present study still has some unavoidable limitations. First, despite assessor-blinding, patients cannot be blinded due to the nature of EA manipulation, which may lead to the occurrence of performance and detection biases. Second, as this study is intended as a pilot study for further larger clinical studies, sample size is another limitation.

In conclusion, this study will provide solid evidence of the role of EA-ACR in the recovery of ULMD of IS patients.

## Author contributions

**Conceptualization:** Gongwei Jia.

**Data curation:** Yilin Liu, Liping Zhang, Qianwen Zang.

**Formal analysis:** Gongwei Jia.

**Funding acquisition:** Gongwei Jia.

**Investigation:** Yilin Liu, Liping Zhang, Lu Long.

**Methodology:** Liping Zhang, Qianwen Zang.

**Project administration:** Sanrong Wang.

**Resources:** Lu Long.

**Software:** Sanrong Wang.

**Supervision:** Liping Zhang.

**Validation:** Qianwen Zang.

**Visualization:** Yilin Liu.

**Writing – original draft:** Yilin Liu.

**Writing – review & editing:** Gongwei Jia.
